# Editorial: Current trends in the applications of bioluminescence

**DOI:** 10.3389/fchem.2023.1309070

**Published:** 2023-10-23

**Authors:** Lijuan Wang, Xixue Lu, Jinxiang Han

**Affiliations:** ^1^ College of Traditional Chinese Medicine, Shandong University of Traditional Chinese Medicine, Jinan, China; ^2^ Neck-Shoulder and Lumbocrural Pain Hospital of Shandong First Medical University, Shandong Academy of Medical Sciences, Jinan, China; ^3^ NHC Key Laboratory of Biotechnology Drugs, Biomedical Sciences College, Shandong First Medical University, Shandong Academy of Medical Sciences, Jinan, China

**Keywords:** editorial, biophoton, traditional Chinese medicine, bioluminescence imaging, non-invasive diagnostic instrument

The field of biophoton is expanding at an accelerated rate. Biophoton is one of the most prominent characteristics of biological organisms. Decades have been devoted to the exhaustive study of biophoton, with researchers focusing on elucidating the mechanisms underlying its generation and investigating its diverse properties. Biophoton, as a promising non-invasive diagnostic instrument, possesses substantial potential and utility for the examination of complex biological systems. Biophoton has been linked to electron transfer processes and the production of reactive oxygen species within the metabolic framework for many years. Its initial application, entrenched in the evaluation of the quality and safety of Chinese herbs, marked a significant turning point. Currently, biophoton encompasses diverse fields, including biology, clinical medicine, chemistry, and traditional Chinese medicine. However, the scope of diseases currently under investigation remains limited, and exhaustive data are urgently needed to strengthen the foundation of biophoton research. Researchers are simultaneously weaving a tapestry of omics techniques and biophoton to decipher the complex theories and mechanisms governing the physiology and pathology of organisms. This convergence gives disciplines including traditional Chinese medicine and clinical medicine new vitality.

Bioluminescence Imaging (BLI) has been developed by expanding upon the foundation of biophoton research and its practical applications. BLI is a cutting-edge technique used to visualize physiological processes in animals, bridging a significant void in the non-invasive monitoring of experimental animals. It enables the observation of multiple physiological processes in real time without necessitating euthanasia, including the non-invasive surveillance of the regulation of islet beta cell function. BLI has the potential to considerably advance pharmacological and physiological research. In recent years, the limitations of single-modal imaging have been addressed by the development of multimodal imaging technology. As an optical imaging technique, BLI provides non-invasive, real-time imaging that precisely identifies the locations and physiological processes of specific cells as well as their interactions with adjacent cells. BLI has predominantly been a preclinical technology, but it has the potential to become a clinical tool for diagnosing and treating human diseases, despite substantial obstacles.

In order to maintain a clinical focus and enhance our involvement in basic science, metabolism, and other clinically relevant areas, the compilation of new research findings in this field will undoubtedly contribute to its future development. We look forward to collaborating with you in the sphere of biophoton in the future years.

